# Pulse Rate Variability Analysis During Hemorrhage in an Experimental Porcine Model

**DOI:** 10.18103/mra.v13i6.6703

**Published:** 2025-06-30

**Authors:** Gabriel P. Bonvillain, Lauren D. Crimmins-Pierce, Md Abul Hayat, Adria Abella Villafranca, Sam E. Stephens, Luke E. Ferguson, Hanna K. Jensen, Joseph A. Sanford, Jingxian Wu, Kevin W. Sexton, Morten O. Jensen

**Affiliations:** 1 Department of Biomedical Engineering, University of Arkansas, Fayetteville, Arkansas; 2 Department of Electrical Engineering, University of Arkansas, Fayetteville, Arkansas; 3 Department of Biomedical Informatics, University of Arkansas for Medical Sciences, Little Rock, Arkansas; 4 Department of Surgery, University of Arkansas for Medical Sciences, Little Rock, Arkansas; 5 Department of Anesthesiology, University of Arkansas for Medical Sciences, Little Rock, Arkansas

## Abstract

**Background:**

The initial response to hemorrhage is mediated by the autonomic nervous system. A method for detecting autonomic nervous system activity may provide the ability to detect acute changes in blood volume. Previous work has indicated that heart rate variability may be able to detect acute hemorrhage in a porcine model. This study evaluates the ability of pulse rate variability, used as a surrogate for heart rate variability, and obtained from peripheral arterial pressure waveforms, to detect hemorrhage in a porcine model.

**Methods:**

Using a model consisting of four subjects, peripheral arterial pressure waveforms were collected over 15-minute intervals before, during, and after hemorrhage. Percent changes for pulse rate variability metrics were evaluated for the 15-minute signals. These longer signals were subsequently divided into three 5-minute signals for short term pulse rate variability analysis. A One-Way Analysis of Variance with a significance level of 0.05 was used to evaluate the sensitivity of pulse rate variability metrics for detecting hemorrhage.

**Results:**

At the onset of hemorrhage, an increase in standard deviation, root mean squared error, low frequency power, high frequency power, total power, and the ratio of low to high frequency power was observed, followed by an opposing decrease at the conclusion of hemorrhage. The low frequency power and the ratio of low to high frequency power had the greatest sensitivity to detecting changes in hemorrhage levels.

**Conclusion:**

These results suggest that pulse rate variability metrics obtained from arterial pressure waveforms have the potential to serve as a diagnostic method for acute hemorrhage.

## Introduction

Traumatic injury is the leading cause of death among individuals under 44 years old^1^. Approximately 20% to 40% of deaths following traumatic injury occur after hospital admissions, and these are potentially preventable with rapid hemorrhage control ^2^. Early detection and accurate diagnosis of hemorrhage is essential to effective treatment. This relies on physical examination and traditional vital signs such as heart rate, arterial oxygen saturation, and blood pressure. Currently, these methods are insensitive to acute changes in blood volume and can remain normal until approximately 1 to 1.5 L of blood loss^2–5^.

The initial physiological response to hemorrhage is characterized by a progressive increase in sympathetic vasoconstriction, peripheral resistance, and heart rate to maintain arterial blood pressure at approximately normal levels^5–8^. This initial response is controlled by the autonomic nervous system (ANS), therefore, a method of measuring the ANS activity has been proposed as a technique for acute hemorrhage detection.

Heart rate variability (HRV) is a signal composed of the beat-to-beat intervals of the heart, which has been identified as a tool for assessing ANS activity^9^. Studies have begun utilizing HRV as a method for assessing the autonomic response to hemorrhage^10,11^; however, heart rate variability measurements are traditionally applied to long-term (24-hour) electrocardiogram (ECG) signals, which limits utility. Recent advancements have proposed the use of shorter time frames (5–20 minutes) as a method for detecting the acute changes in sympathetic innervation^12,13^. Additionally, a method of pulse rate variability (PRV) that utilizes a continuously acquired pulse or pressure waveforms as a surrogate for the ECG recordings traditionally used in HRV measurements has been proposed^13–15^. The use of PRV as a surrogate for HRV provides a significant advantage due to the low cost, and widely accessible methods of acquiring pulse rate variability metrics^16^.

The arterial pressure waveform can identify rapid changes in systolic, diastolic, and mean arterial pressures, so it has been proposed as a way to detect acute hemorrhage^17^. Recent studies have utilized the peripheral arterial pressure waveform to identify changes in pulse wave velocity and pulse wave reflection that are associated with arterial stiffness and hemorrhage-induced vasoconstriction^18,19^. Furthermore, studies have shown that the heart rate can be reliably derived from arterial pressure waveforms^19,20^.

With the increasing utility of continuously acquired arterial pressure waveforms, this study chose to use a porcine model to evaluate the utility of short-term PRV metrics derived from peripheral arterial pressure waveforms as a method for detecting hemorrhage. The goal of this study is to characterize the changes of the short-term PRV metrics while undergoing hemorrhage. While Salomão et al. has previously conducted research on HRV metrics in a porcine model undergoing hemorrhage, prior research on the specific utilization of PRV as a surrogate to HRV during hemorrhage in a porcine model is extremely limited^11^. We hypothesize that PRV metrics obtained from the peripheral arterial pressure waveform during hemorrhage mimic trends in HRV metrics previously reported during hemorrhage by Salomão et al.^11^. Specifically, we expect the frequency domain metrics to experience a significant, positive percent change from the before hemorrhage to during hemorrhage states. Further, we expect metrics in the time domain to experience a significant, positive percent change in a similar fashion. Additionally, we predict that the changes in the PRV metrics previously stated at the onset of hemorrhage will oppose the changes in the PRV metrics at the end of hemorrhage. Lastly, we hypothesize that the use of pulse rate variability metrics obtained from a peripheral arterial pressure waveform will be able to definitively differentiate between hemorrhage statuses.

## Methods

### STUDY POPULATION

Arterial pressure waveforms were obtained from four healthy female porcine subjects under general anesthesia (16–17 weeks old, average age: 16.8 weeks old, average weight: 72.8 ± 1.6 kg).

### INSTRUMENTATION AND SIGNAL ACQUISITION

Each subject was anesthetized with isoflurane at a 1.5% minimum alveolar concentration and propofol infusion at a concentration of 0.05 mg/kg/min prior to instrumentation. The femoral artery was accessed via the left leg. The femoral arterial pressure signal was collected by an MLT0670 disposable blood pressure transducer (ADInstruments). The pressure transducer was connected by MLAC11 Grass adapter cables (ADInstruments) to an FE221 bridge amplifier (ADInstruments). The output from the amplifier was then connected to a USB-6009 data acquisition system (National Instruments) by BNC-to-BNC cables and BNC breakout connectors. This data acquisition device, then interfaced with LabVIEW (National Instruments) to record the pressure waveform with a sampling frequency of 1000 Hz. This sampling frequency was chosen based on the requirements of the hardware used for signal acquisition and processing.

After instrumentation, a baseline 15-minute recording was collected prior to hemorrhage. Continuous 15-minute recordings were taken as each subject was exsanguinated at a rate of 0.4 cc/s from the arterial sensor’s stopcock. The rate of blood loss was determined as an appropriate rate to mimic hemorrhage in this study based on prior porcine hemorrhage models^11^. Approximately 20% blood volume was removed from each subject during the 15-minute window. The amount of blood loss for each subject was determined based on their initial weights. After the hemorrhage concluded, a 15-minute recording was collected during the recovery phase.

### SIGNAL ANALYSIS AND POST PROCESSING

**Figure 1:**
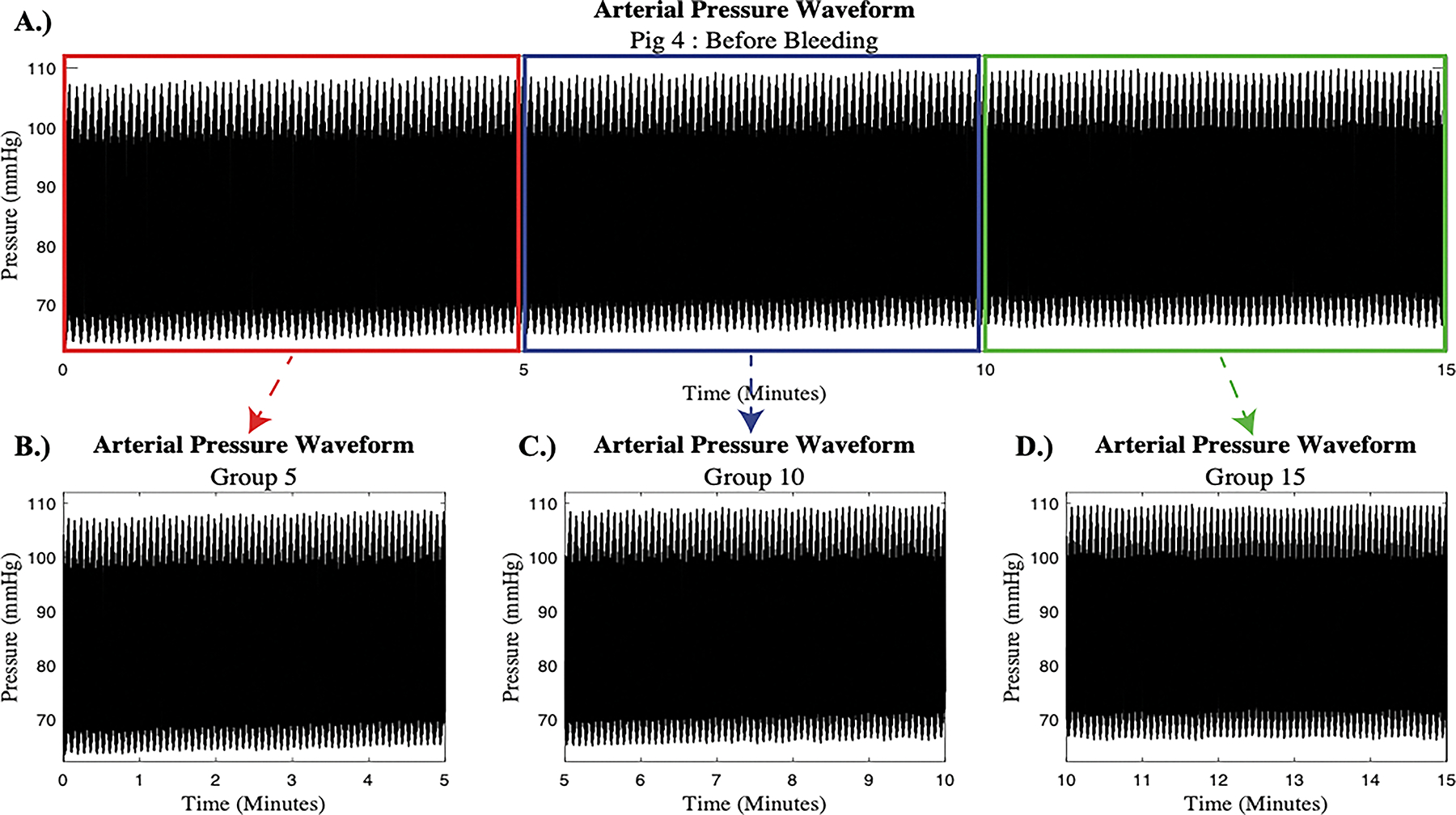
Arterial Pressure Signal: A.) The full 15-minute arterial pressure signal split into three 5-minute signals corresponding to: B.) the first 5 minutes (Group 5, red box), C.) the second 5 minutes (Group 10, blue box), and D.) the last 5-minutes of the 15-minute signal (Group 15, green box).

The collected femoral arterial pressure waveforms were passed through a low-pass filter to exclude noise from other instruments in the room. Prior studies have indicated that due to the low frequency nature of PVP signals, only signal sections within the frequency range from 0 to 20 Hz may be utilized for processing^21^. Therefore, a normalized frequency of 15 Hz was deemed appropriate for the low-pass filter in this study. Each 15-minute signal was preserved to allow for interpretation based on major trends in the PRV signal and then copied and divided into three smaller 5-minute signals for short term PRV analysis. A representative example from subject 4 is depicted in Figure 1.

**Figure 2:**
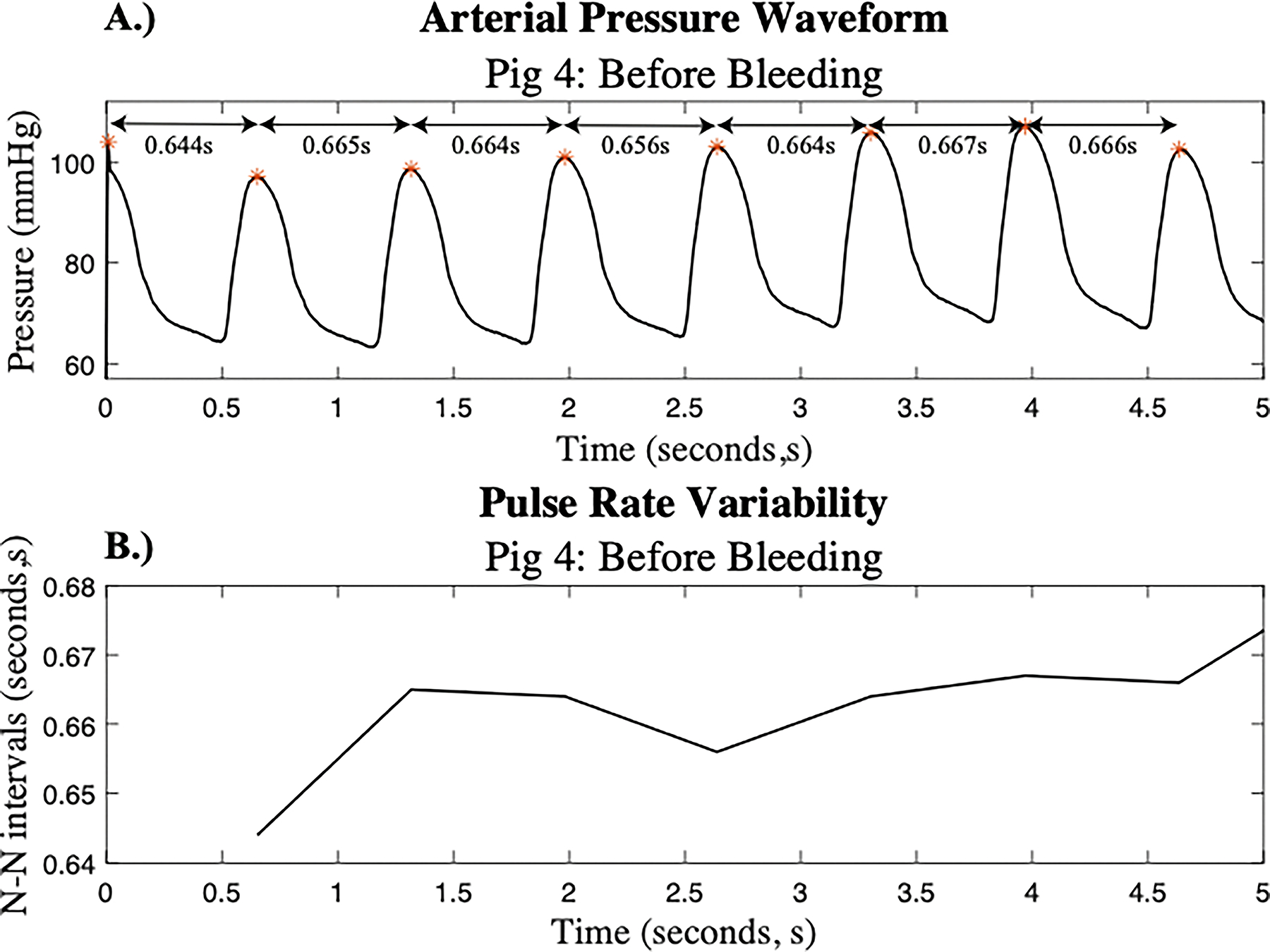
Selected Arterial Pressure Waveform: A.) The arterial pressure waveform from subject 4 prior to hemorrhage with the time (in seconds, s) between beats indicated. B.) The corresponding PRV signal derived from the pulse intervals labeled in A.).

A beat-detection algorithm was then utilized to determine the location of each pulse in the filtered 15-minute signals. The time difference between each pulse, or the pulse ‘peak’ difference, denoted as the P-P interval, was extracted for each signal. An adaptive filter was created to detect and remove abnormal beats and noise disturbances ^22^. This filter was applied to the P-P intervals to create a normal-to-normal beat interval, denoted as the N-N interval. The filtering criteria for the P-P intervals - specifically, the exclusion of intervals shorter than 20 milliseconds or those exceeding three standard deviations from the moving average - were selected to ensure physiologically valid PRV signals. Intervals shorter than 20 milliseconds are not compatible with normal cardiac physiology in porcine subjects and are likely to represent artifacts, such as noise or false peak detection. Similarly, P-P intervals that deviate significantly from the local average often reflect motion artifacts, transient signal loss, or ectopic beats, rather than true autonomic modulation. By removing these non-physiological data points, the resulting N-N interval series more accurately reflects normal beat-to-beat variation driven by autonomic nervous system activity and improves the reliability of derived PRV metrics, both in the time and frequency domains. This approach aligns with established practices in HRV analysis and helps preserve the physiological interpretability of PRV signals. Figure 2 depicts the beat detection used and the corresponding N-N interval that was created for subject 4.

### PULSE RATE VARIABILITY ANALYSIS

Using time domain analysis methods, three PRV indices were calculated. The metrics chosen were derived from the standards of measurement guidelines for HRV indices from the Task Force of the European Society of Cardiology and the North American Society of Pacing and Electrophysiology^9^. The time domain PRV indices that were utilized in this study are the mean N-N interval in milliseconds (Mean), the standard deviation of N-N intervals for the entire signal in milliseconds (STD), and the root mean squared difference between successive N-N intervals in milliseconds (RMS). The power spectra of the PRV signals were taken to determine four PRV indices in the frequency domain. The frequency domain PRV indices that were utilized in this study are the power of the low frequency band, from 0.04–0.15 Hz, in milliseconds squared (LF), the power of the high frequency band, from 0.15–0.4 Hz, in milliseconds squared (HF), the total power of the power spectra in milliseconds squared (P), and the ratio between LF and HF (LF/HF).

### STATISTICAL ANALYSIS

For each porcine subject the percent change from before hemorrhage to during hemorrhage and during hemorrhage to after hemorrhage was assessed for each PRV metric obtained from the 15-minute signal. Then, a one-way analysis of variance (ANOVA) with a significance level of 0.05 was conducted for all PRV metrics obtained from the 5-minute signals to determine if a statistical difference was present between the average of the four subjects. In the one-way ANOVA, groups 5, 10, and 15 correspond to the 5-minute intervals prior to hemorrhage, groups 20, 25, and 30 correspond to the 5-minute intervals during hemorrhage, and groups 35, 40, and 45 correspond to the 5-minute intervals after hemorrhage. The normality of the data was tested with a q-q plot as well as the Anderson-Darling normality test^23,24^. Post-hoc analysis of a pairwise ANOVA was conducted for every possible pair of groups with a p-value of <0.05 to determine if a statistical difference was present between groups. Tukey’s Honestly Significant Difference (HSD) test was applied to correct for multiple comparisons and maintain the family-wise error rate at 0.05.

## Results

### 15-MINUTE ANALYSIS

The percent change in mean, STD, RMS, LF, HF, P, and the LF/HF ratio for the 15-minute recordings before hemorrhage to during hemorrhage across all four porcine subjects are reported in Table 1.

From the 15-minutes before hemorrhage to the 15-minutes during hemorrhage, the percent change in mean N-N intervals increased across three subjects and decreased for one subject. The time domain metrics revealed that the standard deviation and root mean squared deviation of N-N intervals during the 15-minute signals had a positive percent change at the onset of hemorrhage among all subjects. There was a large, positive percent change across all subjects for LF, HF, P, and the LF/HF ratio from before hemorrhage to during hemorrhage.

The percent change in mean, STD, RMS, LF, HF, P, and the LF/HF ratio for the 15-minute recordings during hemorrhage to after hemorrhage across all four porcine subjects are reported in Table 2.

From the 15-minutes during hemorrhage to the 15-minutes after hemorrhage, The percent change in mean N-N intervals of the 15-minute signals continued to increase across three subjects and decrease for one subject. The time domain metrics revealed that the standard deviation and the root mean squared deviation increased for two of the subjects and decreased for two of the subjects. There was a negative percent change, much smaller in magnitude than the positive percent change observed in the 15 minutes before hemorrhage to the 15 minutes during hemorrhage, in LF, P, and LF/HF across all subjects. HF had a positive percent change for one subject and a negative percent change for three subjects.

### 5-MINUTE ANALYSIS

**Figure 3:**
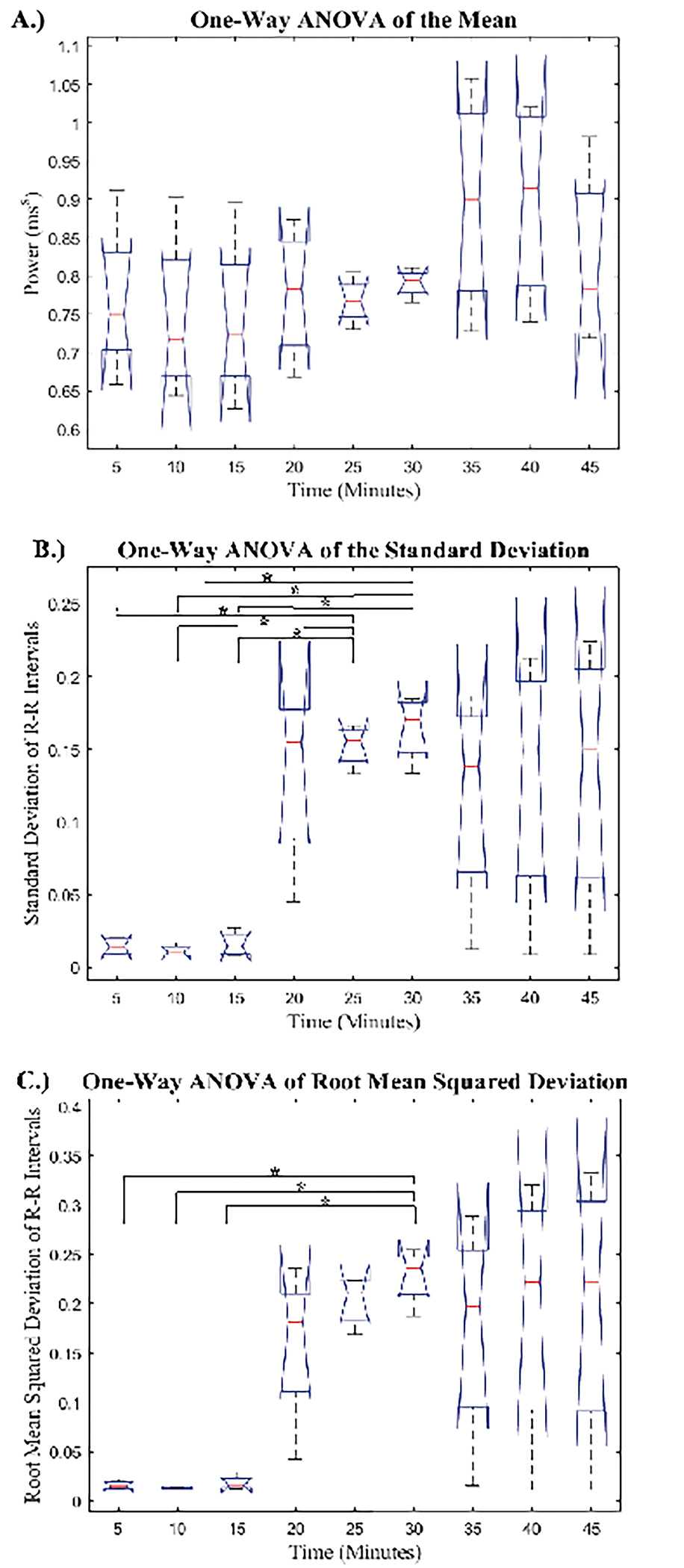
Boxplot Comparison of the Time Domain Metrics for the Nine 5-minute Signals: Boxplot comparison of the mean, standard deviation, and root mean squared deviation from the nine 5-minute signals. Groups 5, 10, and 15 correspond to the 5-minute intervals prior to hemorrhage, groups 20, 25, and 30 correspond to intervals during hemorrhage, and groups 35, 40, and 45 correspond to intervals after hemorrhage. Significant differences are labeled:‘*’

A one-way ANOVA was used to compare the PRV metrics across all nine 5-minute signals. The boxplot comparison of the time domain PRV metrics across each of the 5-minute segments is depicted in Figure 3.

**Figure 4:**
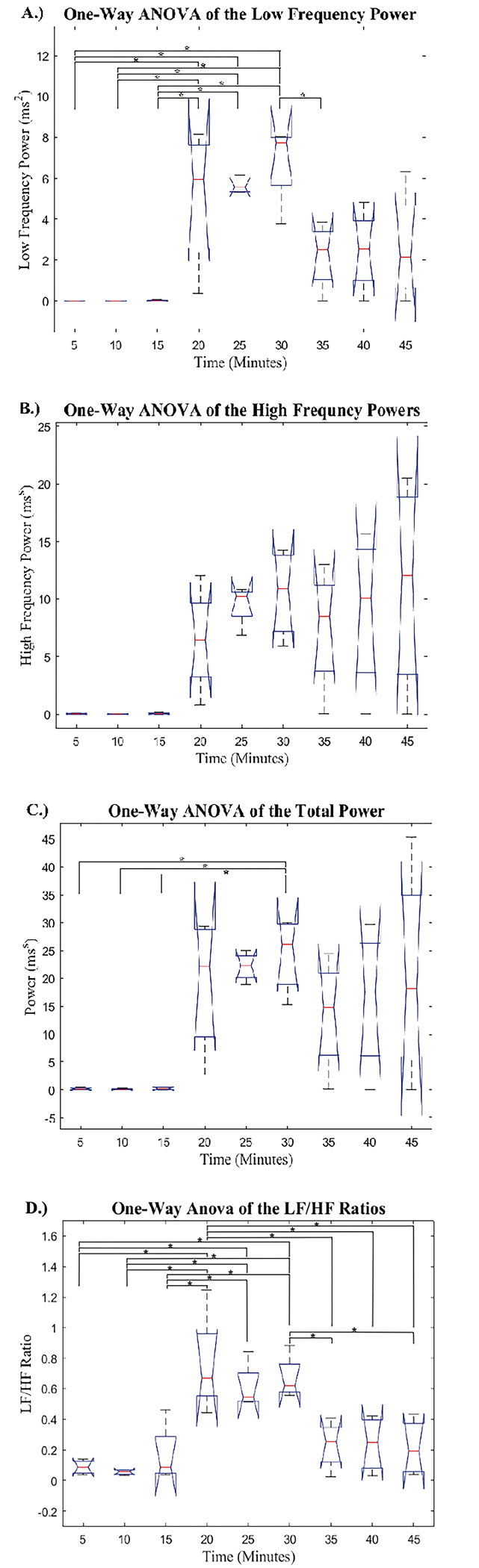
Boxplot Comparison of Frequency Domain Metrics for the Nine 5-minute Single”: Boxplot comparison of the low frequency power, high frequency power, total power, and the ratio between low and high frequency from the nine 5-minute signals. Groups 5, 10, and 15 correspond to the 5-minute intervals prior to hemorrhage, groups 20, 25, and 30 correspond to intervals during hemorrhage, and groups 35, 40, and 45 correspond to intervals after hemorrhage. Significant differences are labeled:‘*’

The mean N-N intervals had no significant difference across all hemorrhage levels. The STD and RMS values obtained from the 5-minute PRV signals showed an increase at the onset of hemorrhage, followed by two progressively smaller increases as hemorrhage continued. Additionally, there was a decrease in both STD and RMS from the last 5-minutes during hemorrhage to the first 5-minutes after hemorrhage. The p-values derived from the post-hoc pairwise comparisons are reported in a table as supplemental digital content (SDC). The pairwise comparisons of the mean, STD, and RMS are reported in SDC1, SDC2, and SDC3, respectively. Post-hoc analysis revealed a significant difference in the RMS values obtained in group 30 as compared to the recordings from baseline (groups 5, 10, and 15). Post-hoc analysis of the STD values determined that groups 25 and 30 were significantly different than the three baseline recordings (groups 5, 10, and 15), which shows greater sensitivity than the mean and RMS metrics. Furthermore, none of the groups after hemorrhage were significantly different than any groups during hemorrhage for both the STD and RMS values. The boxplot comparison of frequency domain metrics for the nine 5-minute signals is displayed in Figure 4

The frequency domain metrics all showed similar trends with an increase at the onset of hemorrhage, followed by smaller increases as hemorrhage progressed. Then, when hemorrhage ended, the PRV metrics displayed a decrease that opposes the trends at the beginning of hemorrhage. The p-values from post-hoc pairwise comparisons for LF, HF, P, and LF/HF are reported in SDC4, SDC5, SDC6, and SDC7, respectively. The post-hoc analysis revealed that the HF values had no significant differences between any of the nine 5-minute signals. Additionally, the P value during the last 5-minute signal during hemorrhage (group 30) was significantly different than all 5-minute baseline recordings (groups 5, 10, and 15), but no groups after hemorrhage had significantly different P values than any groups during hemorrhage. The post-hoc analysis from the one-way ANOVA revealed that the LF values for all groups during hemorrhage (groups 20, 25, and 30) were significantly different than the groups during baseline recording (groups 5, 10, and 15). Additionally, it was found that the final group during hemorrhage (group 30) had significantly different LF values than the first group after hemorrhage ended (group 35). Finally, the analysis of the LF/HF ratio across all nine 5-minute signals displayed a significant difference between all groups during hemorrhage (groups 20, 25, and 30) and the baseline groups prior to hemorrhage (groups 5, 10, and 15). Further, it was found that the first group during hemorrhage (group 20) was significantly different than all the groups after hemorrhage (groups 35, 40, and 45) and the last group during hemorrhage (group 30) was significantly different than two groups after hemorrhage (group 35 and 45).

## Discussion

Currently, precision medicine is not used for the detection and diagnosis of hemorrhage, instead clinicians rely on physical examination and traditional vital signs such as heart rate, arterial oxygen saturation, and blood pressure, which remain at near-normal levels until approximately 1.0 to 1.5 L of blood loss^3–5^. At 20% to 30% blood loss, the second phase of hemorrhage, patients experience insufficient oxygen delivery to vital organs, leading to hemorrhagic shock^5–8^. Hemorrhagic shock is one of the leading early causes of death and has been identified as a predictor of poor outcomes in injured patients ^25^. Therefore, this investigation sought to lay the groundwork to decrease adverse effects of traumatic injury and decrease mortality rates associated with hemorrhage by using algorithmic detection of hemorrhage prior to the decompensatory phase.

Across all PRV metrics, the full 15-minute analysis showed a greater ability to detect the onset of hemorrhage than the conclusion of hemorrhage. All four frequency domain metrics showed a large positive percent change from before hemorrhage to during hemorrhage, which aligned with our expectations and previously reported trends in HRV^11^. This suggests that these metrics have the potential to distinguish between signals during baseline and acute hemorrhage. In the time domain, the standard deviation and root mean squared deviation showed similar results by having a large positive percent change from before hemorrhage to during hemorrhage, which followed the previously reported trends in HRV metrics at the onset of hemorrhage ^11^. This suggests that both the standard deviation and root mean squared deviation have the potential to detect the onset of hemorrhage. Finally, the mean increased across three of the four subjects from before hemorrhage to after hemorrhage. These results oppose the findings of Salomão et al., who found that the mean derived from HRV metrics decreased at the onset of hemorrhage. This suggests that further studies need to be conducted to determine if the mean derived from PRV analysis has the least sensitivity to detecting the onset of hemorrhage.

The observed differences between PRV and HRV trends may be attributed to physiological and signal acquisition disparities. While both metrics reflect autonomic modulation of cardiovascular function, PRV is derived from peripheral vascular pressure waveforms, which are influenced not only by cardiac output but also by vascular compliance, pulse wave transmission, and peripheral resistance. These additional hemodynamic factors can distort the timing and shape of pressure waveforms, particularly during sympathetic activation such as hemorrhage. In contrast, HRV is derived directly from the cardiac electrical cycle via ECG and is less susceptible to these peripheral influences. As a result, PRV may exhibit exaggerated or delayed changes relative to HRV, especially in the frequency domain, where vascular tone and pulse transit time introduce variability not directly related to heart rate modulation.

Analysis of the full 15-minute signals from during hemorrhage to after hemorrhage further emphasized that the frequency domain metrics may have a greater sensitivity to detecting changes in blood volume. For LF, P, and LF/HF there was a negative percent change from during hemorrhage to after hemorrhage for all four porcine subjects. These results opposed the findings from before hemorrhage to during hemorrhage, which suggests that the PRV metrics used may have the ability to identify differences in the onset of hemorrhage and the conclusion of hemorrhage. The magnitude of these percent changes was lower than the magnitude of the percent changes from before hemorrhage to after hemorrhage, which suggests that these metrics may have a greater sensitivity to detecting the onset of hemorrhage than the conclusion of hemorrhage. The HF values had similar results for three subjects but had one subject having opposing results with a positive percent change from during hemorrhage to after hemorrhage. Additionally, the time domain metrics also presented varying results in the full 15-minute analysis from during hemorrhage to after hemorrhage. These opposing trends at the conclusion of hemorrhage may be due to physiological variations in subjects’ ability to recover from blood loss.

The full 15-minute analysis indicates the potential ability for PRV metrics to detect the onset of hemorrhage and, with less sensitivity, the conclusion of hemorrhage. To gain a deeper understanding of the sensitivity of these metrics, we evaluated smaller 5-minute segments of the larger 15-minute signal. The mean and HF metrics showed no significant differences were found across all nine groups of 5-minute signals, which suggests that further methods of analysis may be needed to utilize the mean and HF metrics for detecting differences in hemorrhage statuses. The RMS and P metrics each had the last 5-minute group during hemorrhage being significantly different than all three of the 5-minute groups from prior to hemorrhage. This suggests that these metrics are sensitive enough to detect hemorrhage during the acute phase; however, they were only able to identify hemorrhage after the subject had been hemorrhaging for 10-minutes. Additionally, the 5-minute analysis of STD demonstrated greater sensitivity because the last two groups during hemorrhage were significantly different than all three baseline groups, which shows an ability to detect acute hemorrhage after 5-minutes of hemorrhage. These results indicate that the RMS, STD, and P found from PRV analysis may have the sensitivity to detect the onset of acute hemorrhage. However, none of these metrics had any significantly different groups from during hemorrhage to after hemorrhage, therefore, the 5-minute analysis of these metrics may not have the sensitivity to detect the conclusion of hemorrhage.

5-minute analysis of LF and the LF/HF ratio revealed the greatest sensitivity to detecting the onset of hemorrhage because each group during hemorrhage was significantly different than all three of the baseline recordings prior to hemorrhage. This suggests that these two frequency domain metrics may have the sensitivity to detect acute hemorrhage within the first 5-minutes of hemorrhage and were able to maintain this distinction throughout the entire 15-minutes of hemorrhage. Furthermore, these were the only two metrics that had groups during hemorrhage being significantly different than any groups after hemorrhage concluded, which demonstrates their potential to distinguish between hemorrhage and hypovolemic baseline.

Overall, the significant trends observed in PRV metrics throughout the study provide evidence that PRV-based hemorrhage detection methods may have the ability to aid clinicians as an earlier detection of acute hemorrhage in patients prior to the decompensatory phase, leading to a shorter response time for treatment and therefore a lower patient mortality rate due to hemorrhagic shock.

### LIMITATIONS AND FUTURE DIRECTIONS

The results of this study suggest that PRV metrics may be used as a viable method for assessing the onset and conclusion of hemorrhage; however, this method relies on the sympathetic control over the heart rate. As the heart rate varies under a wide range of environmental and physical stimulation, the trends that we found in this study may be attributed to the increase in heart rate rather than the baroreceptor mediated control of peripheral resistance during hemorrhage. Therefore, future studies should evaluate these metrics under stimulation that changes the heart rate while maintaining constant blood volume to ensure that the changes in PRV metrics found in this study can be attributed to changes in blood volume.

The major limitation to this study is the small sample size. With four subjects, we can identify metrics and trends that may provide the potential to serve as a method for detecting acute hemorrhage, but we are unable to create broad generalizations with the results of this study. Future studies should include a larger number of porcine subjects.

While many studies have identified PRV metrics as a surrogate for HRV, some studies suggest that this may not be completely true. One study found that time domain metrics from PRV had a strong correlation with HRV metrics but showed that frequency domain metrics from PRV do not have the precision to estimate the HRV metrics in the frequency domain^26^. Recently, studies have suggested that the deviation of PRV and HRV frequency domain metrics may be influenced by changing pulse transmittance time and distortions of the pulse wave throughout the vasculature, which is highly influenced by sympathetic activity, peripheral resistance, and cardiac output^27,28^. Additionally, most of these studies rely on photoplethysmography (PPG) signals rather than peripheral venous or arterial pressure waveforms, so future studies should evaluate the comparison between PRV metrics derived from PPG and PRV metrics derived from peripheral venous and arterial pressure waveforms to determine if results found for PRV signals derived from PPG signals may be applied to PRV metrics from these pressure waveforms^16^. Ultimately, further validation of the correlation between HRV and PRV metrics obtained from venous and arterial pressure waveforms is required to determine if PRV obtained in this manner is a reliable surrogate for HRV metrics before using these methods to confer physiological interpretations.

## Conclusion

During the acute phase of hemorrhage, traditional vital signs are insufficient measures for detecting hemorrhage. The results of this study suggest that pulse rate variability metrics obtained from a peripheral arterial pressure waveform has the potential to serve as an indicator of hemorrhage status during the acute phase. Opposing trends in PRV metrics were found at the onset of hemorrhage and at the conclusion of hemorrhage. The results suggest that because the analysis of the frequency domain metrics of the 5-minute signals were found to be the most sensitive to changes in hemorrhage states, 5-minute signals may provide a greater sensitivity to detecting changes in blood volume status than 15-minute signals. Furthermore, it was found that the frequency domain metrics provided a greater sensitivity to the changes in blood volume than the time domain metrics. While the results of this study are promising for the future of early hemorrhage detection, there is a clear need for further research in this area, including expansion of the methods discussed here to a larger sample size and eventual validation in human models.

## Figures and Tables

**Table 1:** Title: Change in the Mean of N-N Intervals From Before Hemorrhage to During Hemorrhage: The percent change in the mean of N-N intervals (Mean), standard deviation (STD), root mean squared deviation (RMS), low frequency power (LF), high frequency power (HF), total power (P), and the ratio between low and high frequency (LF/HF) from before to during hemorrhage for the 15-minute signals.

	PRV Metrics	Pig 1	Pig 2	Pig 3	Pig 4
Time Domain	Mean	2.0%	−10.8%	3.5%	24.5%
	STD	1,344%	1,130%	399%	702%
	RMS	1,494%	1,416%	1,036%	965%
Frequency Domain	LF	644,707%	243,108%	101,639%	25,293%
	HF	39,784%	12,664%	12,534%	12,047%
	P	27,744%	13,359%	2,224%	6,542%
	LF/HF	492%	1,821%	707%	111%

**Table 2:** Title: Change in the Mean of N-N Intervals From During Hemorrhage to After Hemorrhage: The percent change in the mean of N-N intervals (Mean), standard deviation (STD), root mean squared deviation (RMS), low frequency power (LF), high frequency power (HF), total power (P), and the ratio between low and high frequency (LF/HF) from during to after hemorrhage for the 15-minute signals.

	PRV Metrics	Pig 1	Pig 2	Pig 3	Pig 4
Time Domain	Mean	39.9%	−9.0%	32.1%	4.2%
	STD	4.9%	−92.7%	22.7%	−32.6%
	RMS	10.1%	−94.1%	51.6%	−18.3%
Frequency Domain	LF	−64.5%	−100.0%	−58.4%	−60.6%
	HF	−44.4%	−99.5%	91.3%	−13.4%
	P	−57.0%	−99.4%	−4.2%	−58.2%
	LF/HF	−36.2%	−95.8%	−78.3%	−55.2%
